# The knowledge, attitudes, and perceptions toward the Oxford AstraZeneca COVID-19 vaccine amongst Primary Health care workers in North-Central Trinidad

**DOI:** 10.3389/fpubh.2023.1094001

**Published:** 2023-02-06

**Authors:** Raveed Khan, Rachel Albert, Leann Awe, Renee De Four, Tichad Francois, Tahirah Hinds, Avery Kellman, Kelsey Maharaj, Renea Mahon, Chanel Pierre, Alana Ramai, Rameez Baksh

**Affiliations:** Department of Para-Clinical Sciences, The University of the West Indies at St. Augustine, St. Augustine, Trinidad and Tobago

**Keywords:** vaccination, COVID-19 pandemic, knowledge, attitude and practice (KAP), primary care (MeSH)

## Abstract

**Aim:**

To determine the effects of knowledge, attitudes, and perceptions of primary care health workers toward receiving the Oxford AstraZeneca vaccine in North Central, Trinidad.

**Methods:**

A pretested *de novo* questionnaire containing forty-eight (48) closed ended questions and one (1) open ended question was used to gather data. Descriptive and inferential statistics were used to analyze the data obtained from the questionnaire. These included percentages, means and standard deviations for the descriptive aspect and the Chi-Square test to examine any significant associations. Analysis of Variance (ANOVA) was used to assess any significant differences in means among several categories and the independent samples *t*-test for assessing any significant difference in means between two categories.

**Results:**

273 respondents completed the questionnaire. Most of the participants (72.2%) were female and within the age range 25–36 (56.0%). The mean knowledge score about the AstraZeneca vaccine was 16.28 (SD = 2.28) out of 19 with an overall correct response rate of 79%. 30.4% of participants had a good attitude score and 59.7% had a positive perception toward the AstraZeneca vaccine. There were significant associations between knowledge and marital status (*p* = 0.001), income level (*p* = 0.001), education level (*p* < 0.001), and length of employment (*p* = 0.041); attitudes and sex (*p* = 0.01), age (*p* = 0.04), marital status (*p* = 0.009), income level (*p* < 0.001), education level (*p* = 0.005) and category of staff (*p* < 0.001); perception and sex (*p* = 0.002), marital status (*p* = 0.027), income level (*p* < 0.001), and category of staff (*p* < 0.001).

**Conclusions:**

The main contributors to vaccine hesitancy were inadequate duration of clinical trials and fear of adverse side effects. A significant number of participants (17%) were unwilling to get the vaccine due to lack of information.

## Introduction

The COVID-19 pandemic has had major implications worldwide. According to the Ministry of Health—Trinidad and Tobago (MOHTT), in February 2021, there were over 7,600 positive cases locally—including 136 deaths. In Trinidad and Tobago various public health measures were taken to reduce the spread of COVID-19. The key mitigation and containment strategies implemented by the country were evidence-informed and demonstrated an “all-of-government” approach (1). These measures included mask mandates, encouraging social distancing, frequent hand-washing and imposing limitations on the size of public gatherings. One of the most important steps of modern medicine in the prevention of infectious diseases is achieving immunity through Vaccination. Primary Health Care Services in Trinidad and Tobago joined the Global effort to provide vaccines to the population to reduce the burden of the COVID-19 pandemic. The Government of Trinidad and Tobago initially acquired 100,000–120,000 Oxford AstraZeneca COVID-19 Vaccines through the COVID-19 Vaccines Global Access (COVAX) programme (2). These vaccines were initially allocated to high-risk groups including front line health care workers.

Healthcare workers are viewed as reliable sources of information on vaccination as seen in multiple studies, locally and internationally. The success of a vaccination process is greatly determined by healthcare workers' acceptability, knowledge, awareness, and attitudes about COVID-19 vaccination (3). Popa et al. highlighted the role of primary care healthcare professionals, namely, family physicians in promoting vaccine acceptance (4). Locally, De Freitas et al. found that people with high levels of trust in the medical sector were less likely to believe in misinformation (5). Healthcare professionals, therefore, have a significant role in maintaining public trust in vaccination (3).

Furthermore, it has been observed that a negative attitude toward the vaccination process can serve as a Public Health barrier to the achievement of immunity in the population and interventions that address these concerns should be of great importance (6).

The varying attitudes of persons, whether influenced by demographics, ethnicity, educational or social standing, will affect the willingness of vaccination throughout the world. Several studies have been done in multiple countries exploring different attitudes and levels of acceptance of the COVID-19 vaccine among different groups of persons.

In the Democratic Republic of Congo a 27.7% acceptance rate of COVID-19 vaccination amongst healthcare workers has been reported. The willingness of health care workers in Congo toward COVID-19 vaccination was found to be very low when compared with a similar study by Fares et al. in France which revealed that 77.6% of participants “probably agreed” to get vaccinated against COVID-19. The Congo study highlighted that hesitancy is a major barrier to implementation of the vaccine and understanding and addressing vaccine hesitancy is important to maintain the benefits of vaccination programmes (7).

An Israeli study utilizing an anonymous online questionnaire stratified for health care professionals showed that being a healthcare professional did not significantly influence the participants' acceptance of the COVID-19 vaccine; however, doctors working in COVID-19 departments showed higher acceptance rates than those in other departments (8). Furthermore, doctors were generally more accepting of the vaccine than nurses. A significant positive predictor for acceptance of the COVID-19 vaccine was found to be acceptance of the influenza vaccine. This was also reported by Fisher et al. (9).

Safety issues are paramount amongst health care workers. Indeed, the greatest concern to health care workers was the safety of the vaccine with respect to its rapid development, in particular quality control, potential side effects and associated COVID-19 (8, 10).

The following factors were found to be strong predictors of COVID-19 vaccine hesitancy in healthcare workers (6, 8): low-income or unemployed groups, poor adherence to COVID-19 government guidelines, poor perception of disease risk, female gender, and having children.

Overall, a clear understanding of the reasons for vaccine hesitancy is vital in attaining long term control of COVID-19. There is currently no published data related to COVID-19 vaccine perception amongst health care workers. By assessing the healthcare workers' knowledge, attitudes and perceptions toward the Oxford AstraZeneca COVID-19 vaccination, new information becomes available to guide public health initiatives related to vaccine promotion and education.

## Methodology

A cross-sectional study using a three scalar methodology was used to obtain data on the knowledge, attitudes, and perceptions toward the COVID-19 vaccine. A *de novo* questionnaire containing forty-eight (48) closed ended questions and one (1) open ended question was used to gather data. The questionnaire was pre tested during the last 2 weeks of April 2021 and adjustments made to reduce duplications and refine the questions for ease of administration. It was made accessible *via* an online form and completed during the period May 08, 2021 to July 21, 2021. The single open-ended question targeted the address of the participant. Three of the four sections of the questionnaire inquired into the knowledge, attitudes and perceptions toward the Oxford AstraZeneca COVID-19 vaccine respectively and the remaining section addressed participants' demographics (see Appendix 1).

### Sample size calculation

Given the estimated total of 532 Health Care Workers distributed throughout the fifteen (15) primary care facilities within the region, sampling all clinical staff members was determined to be the best way to accurately reflect the KAP of the primary care staff.

For calculating the minimum sample size, the following formula will be used:


$$n_{0}=\frac{z_{\left(1-\alpha \right)}^{2}\hat{p}\left(1-\hat{p}\right)}{D^{2}}$$

Where:

*z*_1−α_ = 1.96 (the value from the standard normal distribution for an error of 0.05).

$\hat{p}\;=$ 0.5 (the estimated prevalence- when unknown as in this case we will use 0.5).

*D* = 0.05 (the margin of error – 5%).

Therefore:


$$\text{Sample size}=\frac{1.96^{2}x\;0.5\left(1-0.5\right)}{0.05^{2}}\;=384.$$

### Sample specification

Target Population: Primary Care Health Care workers employed at institutions in the North Central Region.

Sample selection was Purposive sampling.

### Recruitment methodology

A list of the total number of Health Care workers from primary care facilities within the North Central region was provided by the Regional authority governing the North Central Region, namely the North Central Regional Health Authority (NCRHA).

### Inclusion criteria

Health Care workers employed at primary care facilities within NCRHA over the age of 18 years and consenting to participate.

### Exclusion criteria

i.) Health care workers who refuse to participate.
ii.) Health care workers under the age of 18.

Ethical approval was obtained from the NCRHA.

### Scoring knowledge, attitudes and perceptions

The participants' knowledge was assessed using a total of 13 questions. Four of these questions had a maximum of 2 points, 7 of the questions had a maximum of 1 point and the remaining 2 questions had a maximum of zero. Wrong answers were given a score of zero. The total scores to assess knowledge varies between 0 and 15 points.

The analysis of this section adopted the original Bloom's cut off points (80.0%−100.0%, 60.0%−79.0%, and ≤ 59.0%), which classifies participants into three categories as seen below:

12–15 points—good knowledge, 9–11 points—moderate, < 9—poor.

The second module of the questionnaire assessed the participants' attitudes to the COVID-19 vaccine namely the Oxford AstraZeneca. There was a total of nine (9) questions in this section. Five (5) of these questions were interpreted in one of two ways; answers in support of the COVID-19 vaccine (AstraZeneca) obtaining a score of one and those against the COVID-19 vaccine (AstraZeneca) obtaining a score of zero. Four of the questions had a maximum score of zero.

As such, the total score for this section ranged from 0 to 5 and was then classified into participants with a positive attitude scoring more than or equal to 70% and participants with a negative attitude scoring < 70%.

The third section of the questionnaire focused on the perceptions of the participants toward the COVID-19 vaccine. This was assessed using the Likert scale. There were eight positive statements and two negative statements. A five-point rating scale was used and contained the following categories and points scored for each:

Positive statements: Strongly agree (5 points), Agree (4 points), Neutral (3 points), Disagree (2 points), Strongly disagree (1 point).

Negative Statements: Strongly agree (1 point), Agree (2 points), Neutral (3 points), Disagree (4 points), Strongly disagree (5 points).

The maximum attainable score was 50 points. The tallied score was placed into one of the following categories: positive perception: 38–50 points, neutral perception: 25–37 points, negative perception: < 25 points.

### Statistical methods and software

The Statistical Software for Social Sciences, version 27 (SPSS Inc., Chicago, IL) was used for analyzing the data. Prior to data analysis, the normality was tested using the Kolmogorov—Smirnov and Shapiro—Wilks's test. Furthermore, the internal consistency of the Likert scale used for assessment of Attitude was investigated using Cronbach's alpha. Descriptive and inferential statistics were performed using the Chi-square to test for significant associations, the independent samples t-test and ANOVA were used for comparison of two means and more than two respectively. A *p* < 0.05 was deemed statistically significant.

## Results

From the target population of 384, there were 302 participants of which 273 had complete responses. This gave a response rate of 71% from the sample size investigated. The internal consistency of the Likert scale used for assessment of Attitude was investigated using Cronbach's alpha which gave a result of α = 0.87 indicative of good internal consistency.

### Demographic data

Most of the participants (72.2%) were female and within the age range 25–36 (56.0%). Approximately half of the respondents (49.8%) said they were single and most had tertiary level education (89.7%). The majority were doctors (44.0%) and had been employed over 6 months (86.8%). Other demographic features can be seen in Table 1.

**Table 1:** Demographic features of the participants (*n* = 273).

**Features**		**Frequency (*n*)**	**Percent (*%*)**
Gender	Female	197	72.2
	Male	76	27.8
Age range (year)	18–25	9	3.3
	26–35	153	56.0
	36–45	54	19.8
	46–55	38	13.9
	56–65	18	6.6
	66 and more	1	0.4
Marital status	Common-law marriage	15	5.5
	Divorced	11	4.0
	Married	106	38.8
	Single	136	49.8
	Windowed	5	1.8
Education level	Secondary level	27	9.9
	Tertiary level	245	89.7
	Trade school	1	0.4
Occupation	Dentist	8	2.9
	Dietician	4	1.5
	Doctor	120	44.0
	EMT	1	0.4
	ENA	25	9.2
	PCA—patient care assistant	23	8.4
	Pharmacist	8	2.9
	Registered nurse	52	19.0
	Other	32	11.4
Length of employment	< 6 months	36	13.2
	>6 months	237	86.8

### Source of knowledge about the AstraZeneca vaccine

**Figure 1:**
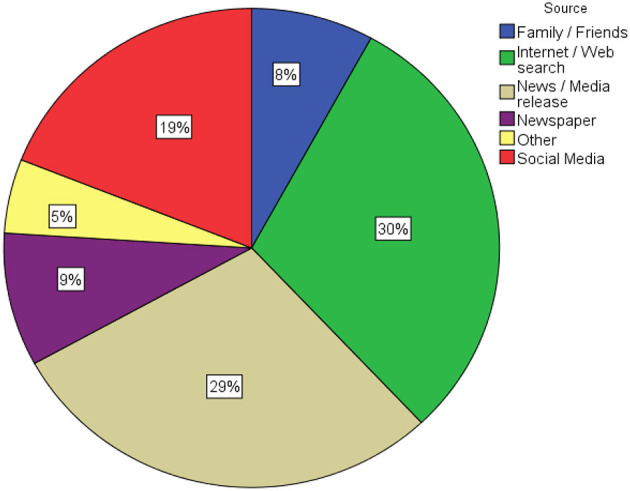
Participants' source of knowledge about the AstraZeneca vaccine.

The mean knowledge score about the AstraZeneca vaccine was 16.28 (SD = 2.28) out of 19 with an overall correct response rate of 79%. Using the Chi-square test, statistically significant associations were seen for knowledge and age (*p* = 0.042) marital status (*p* = 0.001*)*, income level (*p* = 0.001), Education level (*p* < 0.001), NCRHA cluster (*p* = 0.011), category of staff (*p* = 0.016) and length of employment (*p* = 0.041). These results are depicted in Table 2. 186 (68.1%) of the participants had good knowledge, 81 (29.7%) had moderate knowledge and 6 (2.2%) had a poor knowledge score. As seen in Figure 1, the most common source of knowledge about the AstraZeneca vaccine was the internet (30%).

**Table 2:** Participants score by categorization on KAP domains and Chi-square test of association results.

**Variables**	**Knowledge**			***p*-value**	**Attitude**			***p*-value**	**Perception**			***p*-value**
	**Good**	**Moderate**	**Poor**		**Good**	**Moderate**	**Poor**		**Negative**	**Neutral**	**Positive**	
**Sex**												
Male	55	19	2	0.565	25	42	9	**0.01**	2	16	58	**0.002**
Female	131	62	4		58	80	59		15	77	105	
**Age**												
18–25	6	2	1	**0.042**	2	5	2	**0.04**	1	4	4	0.057
26–35	108	43	2		43	77	33		7	51	95	
36–45	38	15	1		17	22	15		2	18	34	
46–55	20	16	2		13	11	14		4	15	19	
56–65	13	5	0		7	7	4		3	5	10	
66 and up	1	0	0		1	0	0		0	0	1	
**Marital status**												
Married	80	26	0	**0.001**	37	38	31	**0.009**	7	37	62	**0.027**
Single	95	38	3		39	74	23		8	37	91	
Divorced	4	6	1		4	2	5		1	7	3	
Common law	6	8	1		1	7	7		0	10	5	
Widowed	1	3	1		2	1	2		1	2	2	
**Income**												
< $5,000	5	3	2	**0.001**	0	5	5	**< 0.001**	1	6	3	**< 0.001**
$5,001–$10,000	57	39	3		14	40	45		14	52	33	
$10,001–$15,000	27	12	0		16	14	9		1	14	24	
$15,001–$20,000	6	1	0		3	4	0		0	0	7	
$20,001–$30,000	85	22	1		49	53	6		0	19	89	
$30,001–$40,000	6	2	0		0	6	2		1	1	6	
>$40,000	0	2	0		1	0	1		0	1	1	
**Education level**												
Secondary level	17	9	1	**< 0.001**	5	8	14	**0.005**	3	12	12	0.26
Trade school	0	0	1		0	0	1		0	1	0	
Tertiary level	169	72	4		78	114	53		14	80	151	
**Category of staff**												
Dentist	3	5	0	**0.016**	4	3	1	**< 0.001**	0	2	6	**< 0.001**
Dietician	1	3	0		0	1	3		0	4	0	
Doctor	96	23	1		50	62	8		0	20	100	
EMT	1	0	0		0	1	0		0	0	1	
ENA	12	12	1		3	6	16		6	15	4	
Other	22	9	0		10	12	9		3	10	18	
PCA	12	9	2		3	7	13		5	12	6	
Pharmacist	7	1	0		1	5	2		0	4	4	
Phlebotomist	0	1	0		0	0	1		0	1	0	
Registered nurse	32	18	2		12	25	15		3	25	24	
**Length of employment**												
< 6 months	18	17	1	**0.041**	7	16	13	0.155	1	17	18	0.169
>6 months	168	64	5		76	106	55		16	76	145	

*p* < 0.05 is statistically significant. Statistically significant values are in bold.

**Figure 2:**
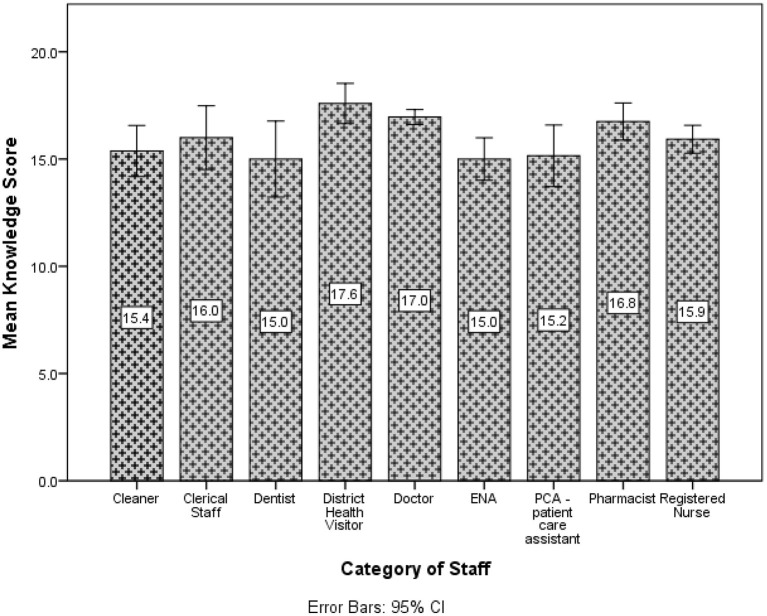
Participants' mean knowledge score about the AstraZeneca vaccine by category of staff.

Doctors, EMTs, District Health Visitors and Veterinarians had a higher average knowledge score compared to the other groups (see Figure 2).

A statistically significant difference in the mean knowledge score (*p* = 0.027) was noted for length of employment. Those working more than 6 months demonstrated higher mean knowledge scores than those employed < 6 months. The ANONA (*p* < 0.001) suggested there was at least one mean knowledge score different for marital status. Upon investigation, there was very strong evidence (*p* < 0.001) of a difference between single persons compared to other groups. Thus, single participants had higher mean knowledge scores than the other groups. Similarly, there was evidence of a difference in the mean knowledge scores for income (*p* < 0.001), age (*p* =0.003), category of staff (*p* < 0.001). The *post-hoc* test indicated this difference occurred between the groups earning < $5,000 and $20,001–$30,000. Participants earning < $5,000 had lower mean knowledge scores, whereas those earning $20,001–$30,000 had higher mean knowledge scores compared to the other categories. The age group 26–35 years and 36–45 years both had higher mean knowledge scores compared to the other groups whereas the age category 46–55 had lower mean knowledge scores compared to the other groups. Doctors had a higher mean knowledge score compared to the other groups.

### Attitude toward the AstraZeneca vaccine

The mean attitude score toward the AstraZeneca vaccine was 2.63 (SD = 1.48) out of 5.83 (30.4%) participants had a good attitude score toward the AstraZeneca vaccine, 122 (44.7%) had a moderate attitude score and 68 (24.9%) had a poor attitude score. Using the Chi-square test, statistically significant associations were seen for attitudes and sex (*p* = 0.01), age (*p* = 0.04), marital status (*p* = 0.009*)*, income level (*p* < 0.001), education level (*p* = 0.005) and category of staff (*p* < 0.001). These results are depicted in Table 2. 186 (68.1%) of the participants had good knowledge, 81 (29.7%) had moderate knowledge and 6 (2.2%) had a poor knowledge score.

**Figure 3:**
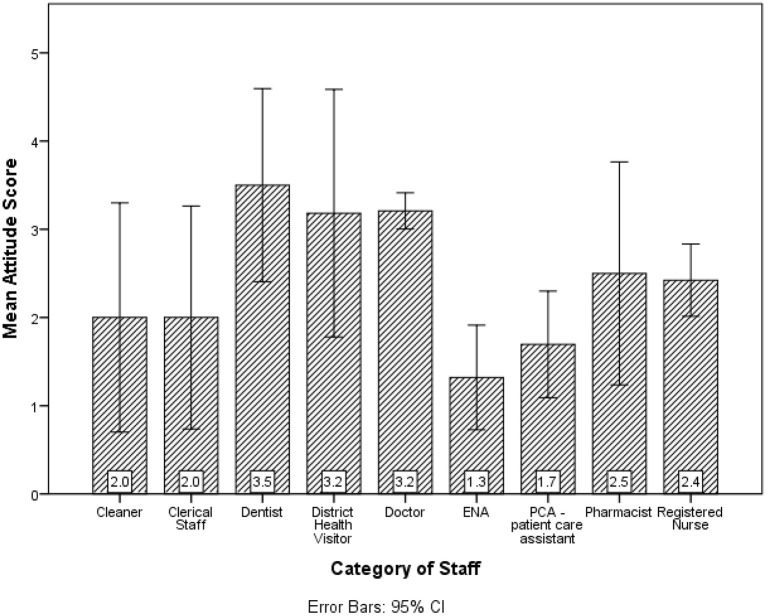
Participants' mean attitude score toward the AstraZeneca vaccine by category of staff.

Dentists had the highest mean attitude scores (see Figure 3). There was a statistically significant difference in the mean attitudes for length of employment (*p* = 0.048) and sex (*p* = 0.031). Those with more than 6 months service had better average attitude scores than those employed for < 6 months. Also, males obtained better average attitude scores than females. The Kruskal-Wallis's test provided very strong evidence of a difference between the mean ranks of at least one pair of groups for marital status (*p* = 0.026), Income (*p* < 0.001), Education level (*p* = 0.004) and category of staff (*p* < 0.001). Upon investigation, there was very strong evidence (*p* < 0.001), single participants had significantly higher mean attitude scores than the other groups whereas participants in common law relationships had lower mean attitude scores. The income categories $15,001–$20,000 and $20,001–$30,000 had higher mean attitudes compared to the other groups whereas < $5,000 had a lower mean attitude score compared to the other groups. Tertiary level had higher mean attitude scores than the other Education categories. Doctors and Dentists had higher mean attitude scores than the other categories of staff.

### Perception of the AstraZeneca vaccine

The mean perception score of the AstraZeneca vaccine was 38.19 (SD = 7.67) out of 45. 17 (6.2%) participants had a negative perception toward the AstraZeneca vaccine, 93 (34.1%) had a neutral perception and 163 (59.7%) had a positive perception. Using the Chi-square test, statistically significant associations were seen for perception and sex (*p* = 0.002), marital status (*p* = 0.027*)*, income level (*p* < 0.001) and category of staff (*p* < 0.001) (see Table 2).

**Figure 4:**
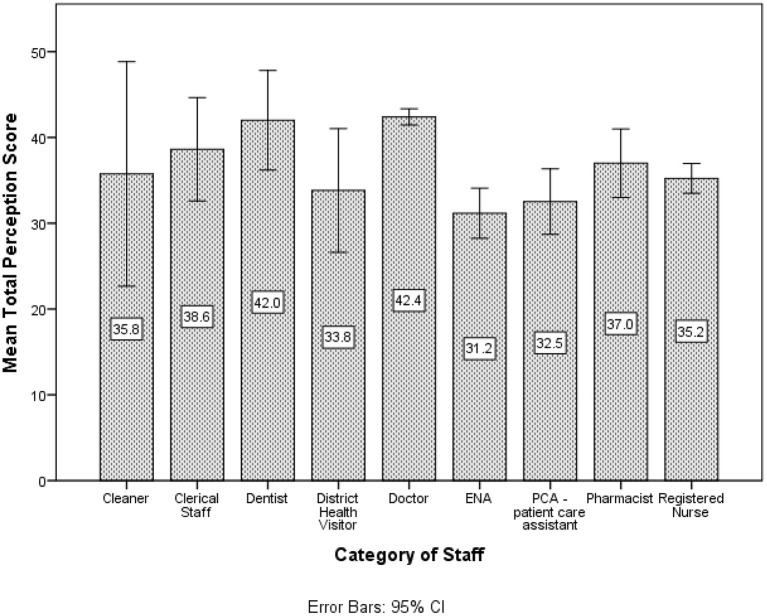
Participants' mean perception score toward the AstraZeneca vaccine by category of staff.

Doctors had the highest average perception score toward the AstraZeneca vaccine (see Figure 4). There was a statistically significant difference in the mean perception score for sex (*p* = 0.001) where males obtained better average perception scores than females. The Kruskal-Wallis's test provided very strong evidence of a difference between the mean ranks of at least one pair of groups for marital status (*p* = 0.049), Income (*p* < 0.001) and category of staff (*p* < 0.001). Upon investigation, there was very strong evidence (*p* < 0.001) that single participants had significantly higher mean perception scores than the other groups. The income categories < $5,000 and $5,001–$10,000 had significantly lower mean perception scores than the other groups. Doctors and Dentists had higher mean attitude scores compared to the other categories of staff whereas Enrolled Nursing Assistants (ENAs) had lower mean perception scores compared to the other categories.

The Chi-square test was used to assess any association between demographic features and categorized scores. Knowledge and Attitude were classified as good, fair, and poor whereas Perception was classified as positive, neutral, and poor (see Table 2).

Statistically significant associations were found between HCW's attitudes and perceptions toward the vaccine (*p* < 0.001) and (*p* < 0.002), respectively (see Table 3).

**Table 3:** Mean scores with respect to demographics and *p* values (independent samples *t*-test for two categories, ANOVA for more than 2).

**Variables**	**Knowledge**		**Attitude**		**Perception**	
	**Mean (SD)**	***p*** **-value**	**Mean (SD)**	***p*** **-value**	**Mean (SD)**	***p*** **-value**
**Sex** ^*^						
Female	16.21 (2.17)	0.812	2.51 (1.54)	**< 0.001**	37.22 (790)	**0.002**
Male	16.41 (2.54)		2.93 (1.25)		40.72 (6.41)	
**Age** ^**^						
18–25	15.22 (3.56)	**0.03**	2.44 (1.51)	0.561	35.89 (7.31)	0.197
26–35	16.55 (2.16)		2.67 (1.34)		38.81 (6.91)	
36–45	16.52 (1.81)		2.59 (1.51)		38.76 (8.52)	
46–55	15.00 (2.67)		2.42 (1.77)		36.32 (8.76)	
56–65	16.31 (2.00)		2.83 (1.82)		35.89 (8.37)	
66 and up	18.50 (0.00)		5.00 (0.00)		47.00 (0.00)	
**Marital status** ^**^						
Common-law marriage	15.00 (3.57)	**< 0.001**	1.53 (1.25)	**0.015**	35.27(6.01)	0.074
Divorced	13.41 (3.23)		2.09 (1.76)		34.00 (8.75)	
Married	16.68 (1.71)		2.67 (1.56)		37.92 (7.84)	
Single	16.43 (2.13)		2.79 (1.34)		39.17 (7.41)	
Widowed	13.70 (3.15)		2.00 (2.00)		35.40 (9.45)	
**Income TTD** ^**^						
< $5,000	13.80 (4.61)	**< 0.001**	1.50 (1.27)	**< 0.001**	34.10 (8.06)	**< 0.001**
$5,001–$10,000	15.76 (2.23)		1.87 (1.43)		33.78 (7.58)	
$10,001–$15,000	16.45 (2.04)		2.97 (1.68)		37.69 (7.25)	
$15,001–$20,000	16.93 (1.92)		3.57 (0.79)		43.14 (3.76)	
$20,001–$30,000	16.90 (1.91)		3.29 (1.09)		42.40 (4.98)	
$30,001–$40,000	16.25 (1.95)		2.25 (0.89)		40.00 (9.12)	
>$40,000	15.00 (0.71)		2.00 (2.83)		35.50 (14.85)	
**Education level** ^**^						
Secondary level	15.69 (2.84)	**0.004**	1.85 (1.43)	**0.003**	35.22 (8.68)	**0.04**
Tertiary level	16.37 (2.17)		2.73 (1.45)		38.56 (7.48)	
Trade school	9.50 (0.00)		0.00 (0.00)		28.00 (0.00)	
**Category of staff** ^**^						
Dentist	15.00 (2.12)	**< 0.001**	3.50 (1.31)	**< 0.001**	42.00 (6.95)	**< 0.001**
Dietician	14.75 (1.50)		0.75 (0.96)		31.00 (5.35)	
Doctor	16.96 (1.93)		3.21 (1.14)		42.39 (5.23)	
EMT	17.00 (0.00)		2.00 (0.00)		42.00 (0.00)	
ENA	15.00 (2.41)		1.32 (1.44)		31.16 (7.08)	
Other	16.50 (1.85)		2.61 (1.61)		37.35 (8.51)	
PCA—patient care assistant	15.15 (3.32)		1.70 (1.40)		32.52 (8.85)	
Pharmacist	16.75 (1.04)		2.50 (1.51)		37.00 (4.78)	
Phlebotomist	15.50 (0.00)		1.00 (0.00)		25.00 (0.00)	
Registered nurse	15.62 (2.34)		2.42 (1.47)		35.23 (6.208)	
**Length of employment** ^*^						
< 6 months	15.61 (2.27)	0.085	2.17 (1.48)	**0.06**	36.94 (6.97)	0.49
>6 months	16.38 (2.27)		2.70 (1.47)		38.38 (7.76)	

*p* < 0.05 is statistically significant.

^*^Independent samples t-test.

^**^ANOVA. Statistically significant values are in bold.

**Figure 5:**
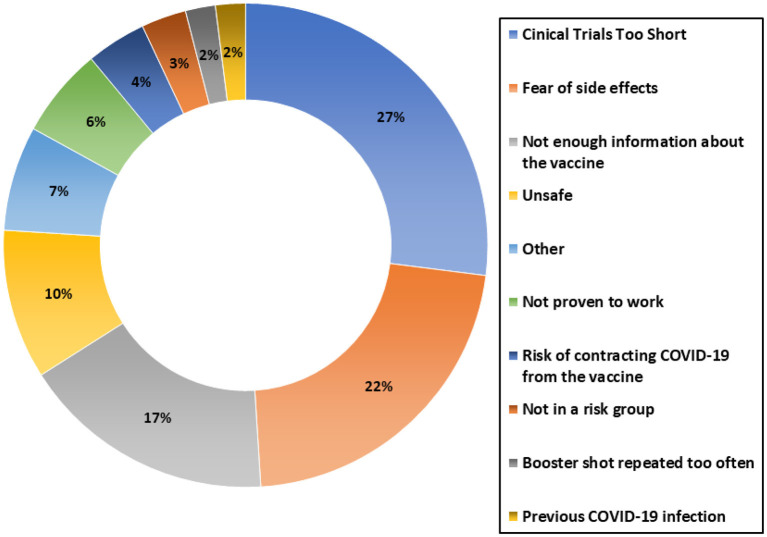
Reasons for not being vaccinated.

The top three reasons for not being vaccinated were, clinical trials being too short (27%), fear of adverse side effects (22%) and not enough information regarding vaccines (17%) (see Figure 5).

## Discussion

### Knowledge

A large portion of staff had good knowledge of the AstraZeneca vaccine. The mean knowledge score was 16.28 (out of 19 as total) with an overall correct response rate to each component of knowledge as 79%. With reference to a participant's knowledge on the AstraZeneca vaccine and their demographic features, many significant findings were discovered.

Single persons between the ages of 26–35 and 36–45, and those employed more than 6 months scored highest in the knowledge category. Furthermore, participants who received tertiary education, and those who made a monthly income more than $20,000TTD had significantly higher knowledge scores. In contrast, those earning a monthly income of < $5,000TTD and attaining only a secondary level education scored lowest in the knowledge domain. These findings are consistent with Islam et al. which showed that participants with higher levels of education and higher socio-economic statuses were more knowledgeable about COVID-19 vaccines (11). Albahri et al. demonstrated that those with higher educational backgrounds and those interested in their health and wellbeing tend to be more knowledgeable on health-based topics (12).

In this study doctors obtained the highest average mean knowledge score compared to other professions. This finding is supported by Mirowsky et al. (13) and Limbu et al. (14) which also reported higher knowledge scores for doctors compared to other HCW's. This is expected as doctors tend to engage in more rigorous research to augment their professional capabilities and may also be more likely to have access to clinical databases and resources (10).

There were significant differences in knowledge amongst various domains; be it age, occupation, marital status, socioeconomic status, or length of employment. It is important however for clinical staff to have minimum baseline levels of knowledge. That way the likelihood of accurate information being disseminated to the public is higher. This is especially important as previous research has documented that the most trusted source of information about the COVID-19 vaccine are from members of the health sector, inclusive of health care workers and the health ministry (5, 12).

In this study the most frequently cited source of information on the COVID-19 vaccine for staff was the internet (30%), followed closely by news or media releases (29%). Indeed, social media is recognized as a significant source of information about the COVID-19 vaccine amongst multiple populations (15, 16). Social media is also thought to provide an overabundance of information which tends to lead to fear and panic and it has also been shown to spread a vast amount of misinformation and contribute significantly to conspiracy theories (17) which concluded that social media would have contributed to a poorer knowledge score.

### Attitudes and perceptions

There were many associations found between demographics and both attitude and perception. Statistically significant associations were found between HCW's attitudes and perceptions toward the vaccine. This is expected as both are contributors to vaccine acceptance. Significant associations were also found between knowledge, attitudes, and perceptions, where they were directly proportional to each other.

Most participants had a moderate to good attitude and a positive perception about the AstraZeneca COVID-19 vaccine. Having a positive attitude and perception was associated with being single, longer lengths of employment, higher incomes, higher education levels and being a medical professional.

Single healthcare workers were more knowledgeable about the COVID-19 vaccine and had higher mean attitude and perception scores. This may be due to the fact that single persons are thought to be more career driven and have more time at their disposal, devoid of domestic commitments (18). Therefore, such persons may be more likely to perform research and stay up to date with current guidelines and recommendations.

No significant associations were found between HCWs age and attitudes or perceptions about the AstraZeneca vaccine. This is at variance with other research findings which demonstrated that older individuals may be more willing to receive a vaccine as they possessed medical experience and perceived COVID-19 as a greater risk (19). In this study, however, it is noteworthy that respondents with a longer length of employment had a better perception of the AstraZeneca vaccine.

Healthcare workers with a lower education level displayed more vaccine hesitancy. Possible reasons for this finding include being less updated on new research, less awareness of the COVID-19 vaccine, greater likelihood of belief in community myths and less concerns of possible risks associated with the COVID-19 virus (19).

Doctors and dentists had the highest mean attitudes and perception scores about the AstraZeneca vaccine. This was in keeping with their higher mean knowledge scores. In addition, attitudes and perceptions were positively correlated to knowledge scores.

A positive association was found between sex, attitudes, and perceptions of the AstraZeneca vaccine. Males had better mean attitude and perception scores compared to females. This finding is consistent with other studies (20, 21). Women are more likely to question the safety, efficacy and quality of the COVID-19 vaccine thereby leading to vaccine hesitancy (22, 23). Vaccine novelty may also help explain this finding. Indeed, newness of the vaccine and a fear of possible adverse effects have been attributed to vaccine hesitancy amongst women (22). Women comprise a large portion of the healthcare workforce. With an increased workload due to the pandemic, they still must balance other responsibilities and if there was an adverse event, they may be unable to perform their abundance of duties (23, 24). It is possible therefore, that the magnitude of the demands on women compounded by the stresses of their jobs contributed to their less favorable attitudes and perceptions to the vaccine (25).

Another factor that is known to contribute to vaccine hesitancy in females is the unknown effects on fertility, pregnancy, and breastfeeding. The COVID-19 virus can have many adverse effects on both mother and fetus. These include preterm labor, pregnancy loss, Intensive Care Unit (ICU) stay and death (24, 26). WHO has since recommended the need for vaccination amongst these populations. Vaccine hesitancy amongst females has also been attributed to misinformation of the effect of COVID-19 on fertility but this is yet to be proven (26).

### Recommendations

Our study revealed that the main contributors to vaccine hesitancy were inadequate duration of clinical trials and fear of adverse side effects. There was also a significant number of participants (17%) who were unwilling to get the vaccine due to lack of information. There are many different strategies that can be used to combat these factors and help in encouraging vaccination.

Greater wide-scale use, and since one of the more prominent and trusted sources of information about the vaccine in this study was news/media releases, informative sessions disseminated *via* the media would likely be beneficial. Infomercials *via* the radio and television with a view to educating the public on vaccine availability and accessibility and highlighting common side effects that can be expected will also assist. Messages can be reinforced by qualified physicians using question-and-answer segments which will assist in promoting accurate information and correcting misinformation.

The following are recommended strategies to promote workplace and community vaccination. Firstly, to be able to get this information to the intended audience, seminars or meetings can be organized which aim to educate and encourage vaccination. These can be online seminars to avoid in-person interactions during this pandemic. Small group sessions of ~10 persons are recommended so that individuals feel comfortable to speak openly and ask questions. These sessions can be led by primary care doctors, or medical experts and can include varying categories of clinical staff per session.

These meetings should follow a general format whereby potential benefits of the vaccine can be discussed, and vaccine confidence enhanced. The aim is to educate by firstly giving key facts on the vaccine, debunking myths, and answering the frequently asked questions inclusive of vaccine exemption eligibility. These meetings can also include other HCWs who have been vaccinated and those who have personal experiences with the COVID-19 virus, who can emphasize the seriousness of the disease, its complications, and the importance of being vaccinated.

Post-seminar, physicians can be made available for the purposes of health promotion and sensitization. Those who are afraid to get vaccinated or believe they should have an exemption but do not fit the criteria can be offered a general check-up to ensure that it is safe for them to take the vaccine and perhaps increase their willingness to receive vaccination.

Another factor that is known to help encourage vaccination is to provide incentives or benefits to vaccinated workers (27). For those who opt to get vaccinated, time off from work can be granted for recovery from possible side effects. Accessories such as vaccination card holders and stickers can also be made available at no cost to further enhance uptake. Confirming appointments in advance for vaccination can assist in reducing wait-times and enhancing the efficiency of the vaccination process. Social media and networks can be utilized to issue reminders for appointments and provide additional information and updates regarding vaccine accessibility and availability.

Our study provides novel findings related to Health Care workers receptivity toward vaccination in our country in the context of a pandemic. It is envisaged that these findings will augur well for pandemic preparedness and response going forward.

### Limitations

The study population was limited to Primary Health care workers in North Central, Trinidad. Ideally, to acquire a national representation of a health care worker's knowledge, attitudes and perceptions toward the AstraZeneca vaccine, the study population should incorporate all the regions within Trinidad and Tobago. Additionally, such a study should be expanded to include staff at Primary, Secondary and Tertiary health care facilities. A follow up study would therefore be encouraged to determine whether the inferences drawn in this study apply nationwide.

Also at the time this study was conducted, the AstraZeneca vaccine was the only vaccine available in Trinidad and Tobago. Ideally, future research should incorporate all brands of COVID-19 vaccines available in Trinidad and Tobago to enable a more accurate representation of the knowledge, attitude, and perceptions toward the COVID-19 vaccine amongst healthcare workers.

It is acknowledged that effect sizes could not be computed due to the nature of the analyses performed and that this study is underpowered as the calculated sample size of 384 was not attained. This limits the generalizability of our findings.

## Data availability statement

The raw data supporting the conclusions of this article will be made available by the authors, without undue reservation.

## Ethics statement

The studies involving human participants were reviewed and approved by North Central Regional Health Authority. The patients/participants provided their written informed consent to participate in this study.

## Author contributions

RK conceived and designed the study and wrote the paper. RB performed the data analysis. RA, LA, RD, TF, TH, AK, KM, RM, CP, and AR collected data and conceived and designed the analysis. All authors contributed to the article and approved the submitted version.
