# Supplementary material for: The knowledge, attitudes, and perceptions toward the Oxford AstraZeneca COVID-19 vaccine amongst Primary Health care workers in North-Central Trinidad
Source: Front Public Health. 2023 Feb 6;11:1094001. doi: 10.3389/fpubh.2023.1094001 (PMC9939478; doi:10.3389/fpubh.2023.1094001)
Supplement: Supplementary file 1 [file Table_1.DOCX]

# APPENDIX 1

Distribution of each Knowledge component and participant responses

| Q11: Do you believe that COVID-19 poses a significant health risk to the population? | ✓ Yes | 257 | 94.1% |
| --- | --- | --- | --- |
|  | ✕ No | 6 | 2.0% |
|  | ✕ Maybe | 10 | 3.7% |
| Q12: What populations do you think are most affected by the COVID-19 virus? Select all that apply. | ✕ Young children | 36 | 13.2% |
|  | ✓ The elderly | 210 | 76.9% |
|  | ✕ Pregnant women | 49 | 17.9% |
|  | ✓ Those with medical conditions | 249 | 91.2% |
| Q13: What are the main ways COVID-19 can be transmitted from an infected person? Select all that apply. | ✓ Respiratory droplets | 255 | 93.4% |
|  | ✓ Infected secretions / on surfaces | 211 | 77.3% |
|  | ✕ Airborne | 177 | 64.8% |
|  | ✕ Food-borne | 9 | 3.3% |
|  | ✕ Sexually transmitted | 24 | 8.8% |
| Q14: What measures can be taken to minimize the spread of COVID-19? Select all that apply. | ✓ Wearing a face mask | 252 | 92.3% |
|  | ✓ Wearing a N95 mask | 226 | 82.8% |
|  | ✓ Wearing double masks | 158 | 57.9% |
|  | ✓ Washing hands often | 263 | 96.3% |
|  | ✓ Sanitizing hands | 257 | 94.1% |
|  | ✕ Sun exposure | 30 | 11.0% |
|  | ✓ Staying 6 feet apart when in public spaces | 258 | 94.5% |
|  | ✓ Staying at home | 215 | 78.8% |
|  | ✓ Staying at home if you have flu-like symptoms | 252 | 92.3% |
|  | ✓ Cleaning / disinfecting frequently touched places | 255 | 93.4% |
|  | ✕ Alcohol consumption | 12 | 4.4% |
|  | ✓ Avoiding social gatherings / large crowds | 255 | 93.4% |
| Q15: Do you think that it is possible to be infected with COVID-19 and not show symptoms? | ✓ Yes | 266 | 97.4% |
|  | ✕ No | 1 | 0.4% |
|  | ✕ Maybe | 6 | 2.2% |
| Q16: Do you think that the Oxford AstraZeneca COVID-19 vaccine is effective? | ✓ Yes | 192 | 70.3% |
|  | ✕ No | 16 | 5.9% |
|  | ✕ Maybe | 65 | 23.8% |
| Q17: If you answered yes to number 16, what percentage effectiveness do you think the vaccine has after completing vaccination? | ✕ 10-30% | 3 | 1.1% |
|  | ✕ 31-50% | 19 | 7.0% |
|  | ✕ 51-80% | 174 | 63.7% |
|  | ✓ 81-100% | 77 | 28.2% |
| Q18: Do you believe that a person can develop COVID-19 after taking the vaccine? | ✓ Yes | 242 | 88.6% |
|  | ✕ No | 10 | 3.7% |
|  | ✕ Unsure | 21 | 7.7% |
| Q19: The Oxford AstraZeneca COVID-19 vaccine is safe for use in which of the following populations? Tick all that apply: | ✕ Young children | 22 | 8.1% |
|  | ✓ The elderly | 260 | 95.2% |
|  | ✕ Pregnant women | 34 | 12.5% |
| Q20: What is the recommended number of doses to achieve optimal immunity using the Oxford AstraZeneca COVID-19 vaccine? | ✕ 1 | 1 | 0.4% |
|  | ✓ 2 | 261 | 95.6% |
|  | ✕ 3 | 2 | 0.7% |
|  | ✕ Unsure | 9 | 3.3% |
| Q21: The COVID-19 vaccine protects you from which of the following? Select the single best answer. | ✕ Contracting COVID-19 virus | 17 | 6.2% |
|  | ✕ From transmitting COVID-19 virus | 250 | 91.6% |
|  | ✕ From re-infection with COVID-19 virus | 0 | 0.0% |
|  | ✓ From serious illness if COVID-19 virus is contracted | 6 | 2.2% |

Distribution of each Attitude component and participant responses

| Q23: Do you usually get your yearly flu vaccine? | ✓ Yes | 135 | 49.5% |
| --- | --- | --- | --- |
|  | ✕ No | 83 | 30.4% |
|  | ✕ Sometimes | 55 | 20.1% |
| Q24: Do you believe that there is enough information being disseminated locally regarding the COVID-19 vaccine? | ✓ Yes | 104 | 38.1% |
|  | ✕ No | 144 | 52.7% |
|  | ✕ Unsure | 25 | 9.2% |
| Q26: Do you believe that the COVID-19 vaccine was developed too quickly? | ✕ Yes | 102 | 37.4% |
|  | ✓ No | 114 | 41.8% |
|  | ✕ Maybe | 57 | 20.9% |
| Q27: If you have not yet received the Oxford AstraZeneca COVID-19 vaccine, will you be willing to be vaccinated? | ✓ I have already been vaccinated/ received the first dose of the vaccine | 171 | 62.6% |
|  | ✓ Yes, I am willing to receive the vaccine | 33 | 12.1% |
|  | ✕ No, I am unwilling to receive the vaccine | 28 | 10.3% |
|  | ✕ Unsure | 41 | 15.0% |
| Q30: Do you believe that the Oxford AstraZeneca COVID-19 vaccine that Trinidad and Tobago have received will be as effective as other brands of the vaccine in decreasing the spread of COVID-19? | ✓ Yes | 152 | 55.7% |
|  | ✕ No | 47 | 17.2% |
|  | ✕ Unsure | 74 | 27.1% |

Distribution of each Perception component and participant responses

| Q32: Vaccines are an effective way to prevent the spread of diseases. | ✕ Strongly disagree | 23 | 8.4% |
| --- | --- | --- | --- |
|  | ✕ Disagree | 7 | 2.6% |
|  | ✕ Neutral | 23 | 8.4% |
|  | ✕ Agree | 67 | 24.5% |
|  | ✓ Strongly agree | 153 | 56.0% |
| Q33: The COVID-19 vaccine will be effective in decreasing the spread of the COVID19 virus. | ✕ Strongly disagree | 14 | 5.1% |
|  | ✕ Disagree | 14 | 5.1% |
|  | ✕ Neutral | 45 | 16.5% |
|  | ✕ Agree | 106 | 38.8% |
|  | ✓ Strongly agree | 94 | 34.4% |
| Q34: The benefits of taking the COVID-19 vaccine outweighs the risks | ✕ Strongly disagree | 14 | 5.1% |
|  | ✕ Disagree | 7 | 2.6% |
|  | ✕ Neutral | 56 | 20.5% |
|  | ✕ Agree | 86 | 31.5% |
|  | ✓ Strongly agree | 110 | 40.3% |
| Q35: There are many serious side effects of the Oxford AstraZeneca COVID-19 vaccine | ✓ Strongly disagree | 32 | 11.7% |
|  | ✕ Disagree | 66 | 24.2% |
|  | ✕ Neutral | 92 | 33.7% |
|  | ✕ Agree | 62 | 22.7% |
|  | ✕ Strongly agree | 21 | 7.7% |
| Q36: Vitamins and herbal supplements are effective in preventing and treating COVID-19 and therefore the vaccine is not necessary. | ✓ Strongly disagree | 129 | 47.3% |
|  | ✕ Disagree | 83 | 30.4% |
|  | ✕ Neutral | 36 | 13.2% |
|  | ✕ Agree | 17 | 6.2% |
|  | ✕ Strongly agree | 8 | 2.9% |
| Q37: As a clinical healthcare worker, I believe that my recommendation of the COVID-19 vaccine will have an impact on a patient's choice to be vaccinated. | ✕ Strongly disagree | 7 | 2.6% |
|  | ✕ Disagree | 11 | 4.0% |
|  | ✕ Neutral | 38 | 13.9% |
|  | ✕ Agree | 124 | 45.4% |
|  | ✓ Strongly agree | 93 | 34.1% |
| Q38: As a healthcare worker, I believe that the COVID-19 vaccine should be mandatory for all frontline workers. | ✕ Strongly disagree | 47 | 17.2% |
|  | ✕ Disagree | 64 | 23.4% |
|  | ✕ Neutral | 60 | 22.0% |
|  | ✕ Agree | 44 | 16.1% |
|  | ✓ Strongly agree | 58 | 21.2% |
| Q39: I will be protecting my family from the COVID-19 virus if I take the vaccine. | ✕ Strongly disagree | 12 | 4.4% |
|  | ✕ Disagree | 21 | 7.7% |
|  | ✕ Neutral | 46 | 16.8% |
|  | ✕ Agree | 108 | 39.6% |
|  | ✓ Strongly agree | 86 | 31.5% |
| Q40: If I take the COVID-19 vaccine, I will be protecting my community from COVID19. | ✕ Strongly disagree | 13 | 4.8% |
|  | ✕ Disagree | 22 | 8.1% |
|  | ✕ Neutral | 49 | 17.9% |
|  | ✕ Agree | 106 | 38.8% |
|  | ✓ Strongly agree | 83 | 30.4% |
| Q41: As a clinical healthcare worker, I would recommend the COVID-19 vaccine to non-healthcare persons such as my family and friends. | ✕ Strongly disagree | 10 | 3.7% |
|  | ✕ Disagree | 7 | 2.6% |
|  | ✕ Neutral | 40 | 14.7% |
|  | ✕ Agree | 82 | 30.0% |
|  | ✓ Strongly agree | 134 | 49.1% |
